# NK-cell-dependent killing of colon carcinoma cells is mediated by natural cytotoxicity receptors (NCRs) and stimulated by parvovirus infection of target cells

**DOI:** 10.1186/1471-2407-13-367

**Published:** 2013-07-31

**Authors:** Rauf Bhat, Jean Rommelaere

**Affiliations:** 1 German Cancer Research Center (DKFZ), Tumor Virology, F010, Im Neuenheimer Feld 242, Heidelberg D-69120, Germany

**Keywords:** Colon carcinoma, Cytotoxicity, Human natural killer cells, IL-2, Oncolytic virus, Parvovirus, Natural cytotoxicty receptors

## Abstract

**Background:**

Investigating how the immune system functions during malignancies is crucial to developing novel therapeutic strategies. Natural killer (NK) cells, an important component of the innate immune system, play a vital role in immune defense against tumors and virus-infected cells. The poor survival rate in colon cancer makes it particularly important to develop novel therapeutic strategies. Oncolytic viruses, in addition to lysing tumor cells, may have the potential to augment antitumor immune responses. In the present study, we investigate the role of NK cells and how parvovirus H-1PV can modulate NK-cell mediated immune responses against colon carcinoma.

**Methods:**

Human NK cells were isolated from the blood of healthy donors. The cytotoxicity and antibody-mediated inhibition of NK cells were measured in chromium release assays. Phenotypic assessment of colon cancer and dendritic cells was done by FACS. The statistical significance of the results was calculated with Student’s *t* test (*p <0.05; **, p < 0.01; ***, p < 0.001).

**Results:**

We show that IL-2-activated human NK cells can effectively kill colon carcinoma cells. Killing of colon carcinoma cells by NK cells was further enhanced upon infection of the former cells with parvovirus H-1PV. H-1PV has potent oncolytic activity against various tumors, yet its direct killing effect on colon carcinoma cells is limited. The cytotoxicity of NK cells towards colon carcinoma cells, both mock- and H-1PV-infected, was found to be mostly mediated by a combination of natural cytotoxicity receptors (NCRs), namely NKp30, 44, and 46. Colon carcinoma cells displayed low to moderate expression of NK cell ligands, and this expression was modulated upon H-1PV infection. Lysates of H-1PV-infected colon carcinoma cells were found to increase MHC class II expression on dendritic cells.

**Conclusions:**

Altogether, these data suggest that IL-2-activated NK cells actively kill colon carcinoma cells and that this killing is mediated by several natural cytotoxicity receptors (NCRs) in combination. Additionally, in association with parvovirus H-1PV, IL-2-activated NK cells have the potential to boost immune responses against colon cancer.

## Background

Natural killer cells constitute a separate lineage of lymphocytes capable of mediating early innate immune responses to viral infections and transformed malignant cells. NK cells can recognize target cells, including tumor cells, having downregulated MHC class I expression. They can also recognize molecules that are not expressed in normal cells but upregulated in transformed cells [1]. NK cells express an array of receptors which modulate their cytotoxicity towards tumor cells and infected cells. These include NK-specific receptors called natural cytotoxicity receptors (NCRs), represented by NKp30, NKp44, and NKp46. NCRs act in concert with other receptors, such as NKG2D and DNAM-1, to mediate NK-cell killing of tumor cells when the corresponding ligands, variably expressed by different tumors, are present. These ligands are upregulated upon transformation and during viral infection [2,3]. The NK receptors involved in killing colon cancer cells have not yet been studied.

Colorectal cancer is the third most common cancer. With an incidence of nearly 1 million cases yearly worldwide, it is the fourth most frequent cause of cancer death [4]. Although advances in colorectal cancer therapy have improved treatment and survival, the 5-year survival rate remains around 50%, making it necessary to develop novel or complementary therapeutic strategies. Colorectal cancer causes multifactorial changes in the host defense system, including loss of HLA class I molecules, impaired NK cell function, and immune escape, resulting in poor prognosis [5]. It is the ambition of immunotherapy to counteract the various escape effects, particularly in early tumors, and to boost immune activity against tumors. To be successful, immunotherapy must overcome immune escape mechanisms and reduce tumor-induced immune suppression. To subdue these impairments, cytokines, particularly IL-2, appear as promising tools for relieving immune suppression and re-activating NK cells.

Novel therapeutic approaches such as virotherapy with oncolytic viruses are also currently pursued as combination strategies. Because oncolytic viruses specifically target and kill tumor cells while sparing normal cells, they represent a promising strategy for combating tumors. Parvoviruses possess both oncolytic and oncosuppressive activities. They are small (25-30 nm), non-enveloped particles housing a 5.1-kb single-stranded DNA genome expressing two major non-structural proteins (NS1 and NS2) and two viral capsid proteins (VP1 and VP2). Epidemiological studies in humans have revealed a correlation between serological evidence of parvoviral infection and a lower incidence of certain cancers [6,7]. Parvoviruses are non-pathogenic in humans, but human tumor cell lines have proven sensitive to the lytic activity of H-1PV *in vitro*. *In vivo*, parvoviruses have been shown to exert oncosuppressive activity against implants of tumor cells, including human neoplastic cells, in recipient animals [8]. Parvovirus H-1PV shows poor oncolytic activity in colon carcinoma models. Colon carcinoma cell lines are sensitive to H-1PV killing, but only at high MOIs and after a long incubation period (8 days). H-1PV infection of colon carcinoma cells leads to production of cytotoxic NS-1 protein, but the ability of the virus to replicate is impaired in colon cancer cells [9,10].

In addition to direct virus-induced oncolysis, an immune component also appears to contribute to parvovirus-mediated oncosuppression. We have previously shown that parvovirus H-1PV, in addition to killing pancreatic cancer cells, can also induce these cells to activate NK cells and boost the innate immune system through enhanced production of IFN-γ and TNF-α [11]. Therefore, oncolytic viruses should be tested further for the ability to augment antitumor immune responses in colon carcinoma. No colon carcinoma model has yet been tested for immune modulation induced by parvovirus-H-1PV.

In the present study we show that IL-2-activated NK cells can effectively kill colon carcinoma cells. We also demonstrate that parvovirus H-1PV infection of colon carcinoma cells can enhance NK-cell-mediated killing of these cells, and that the cytotoxicity of NK cells towards mock- and H-1PV-infected colon carcinoma cells is mediated mostly by a combination of natural cytotoxicty receptors, namely NKp30, 44, and 46. We evidence moderate modulation of NCR ligands in colon carcinoma cells upon infection with H-1PV. Lastly, we show that lysates of H-1PV-infected colon carcinoma cells cause an increase in MHC class II expression on dendritic cells.

In conclusion, our data show that NK cells can kill colon cancer cells and suggest that H-1PV-based immunotherapy might increase NK-cell-mediated immune responses, thus providing a basis for combination therapy against colon cancer.

## Methods

### Cell cultures

Human NK cells were isolated by negative depletion from peripheral blood lymphocytes by means of an NK-cell isolation kit (Dynal, Karlsruhe, Germany). The NK population consisted of 90% - 99% cells displaying the CD3^−^ CD56^+^ phenotype. NK cells were expanded in the presence of IL-2 (100 IU/ml) (NCI-FCRC, preclinical repository) for 5–8 days in RPMI, 10% FCS, penicillin/streptomycin (Invitrogen, Karlsruhe, Germany). They were also obtained by co-culturing peripheral blood mononuclear cells (PBMCs) and irradiated RPMI 8866 cells, as described elsewhere [11]. Alloreactive CD8+ T-cells were isolated from the blood of healthy donors with the Dynabeads Untouched human CD8+ T-cell kit (Invitrogen, Norway) according to the manufacturer’s instructions. They were stimulated with IL-2 (100 IU/ml) for a week. Human DCs were prepared from freshly drawn blood from healthy donors. They were prepared either by the adherence method or with the Dynabeads Untouched human monocyte kit (Invitrogen, Norway) according to the manufacturer’s instructions. For the adherence method, PBMCs were washed twice with phosphate-buffered saline (PBS) and resuspended in X-Vivo 15 medium (BioWhittaker) supplemented with 2 mM L-glutamine, 50 U/ml penicillin, and 50 μg/ml streptomycin. The PBMCs were plated at the density of 6 × 10^6^ cells/ml. After incubation at 37°C for 2 h or overnight, non-adherent cells were removed by washing with PBS. Adherent monocytes were cultured for 6 days in X-vivo 15 medium supplemented with 1000 IU/ml IL-4 (Promokine, Promocell, Heidelberg, Germany) and 500 IU/ml granulocyte macrophage–colony-stimulating factor (Leukine, Uni-apotheke, Heidelberg, Germany). Colo32, SW480, HT29, and Lovo cells were maintained in monolayer cultures under standard conditions (37°C, 5% CO_2_) in DMEM/RPMI medium supplemented with 10% FCS, glutamine, and antibiotics.

### H-1PV infection and lysate preparation

H-1PV was produced by infection of NB-324 K cells. Recombinant H-1PV expressing the marker EGFP (Chi-hH1/EGFP) was obtained by transfection of 293 T cells, as described previously [12]. Virus infections were performed at 37°C for 1 h with a small inoculum of purified virus (wild-type H-1PV or Chi-hH1/EGFP) with occasional rocking of the plate. Mock infection was performed by incubating the cells with FCS-free DMEM medium only. Virus stocks were purified by iodixanol gradient centrifugation and titrated either by plaque assay or by infected cell hybridization assay on NB-324 K indicator cells. Virus titers are expressed in plaque forming units (PFU) or replication units (RU) per milliliter of virus suspension, as described elsewhere [12-14]. For lysate preparation, mock- and H-1PV-infected cells were harvested, centrifuged, and washed with PBS. The pellets were resuspended in PBS and freeze-thawed four times by alternative immersion in liquid N_2_ and a water bath maintained at 37°C, with occasional vortexing. The lysates were then centrifuged at 4°C and 13000 rpm for 30 minutes. The protein content of the lysates was estimated by measuring the absorbance at 280 nM with a Nanodrop instrument.

### FACS analysis and antibodies

The following antibodies were used: anti-CD56 (isotype IgG2a) and anti-CD3 (isotype IgG1, Immunotools, Friesoythe, Germany) coupled, respectively, with FITC or PE (BD Biosciences, Heidelberg, Germany). Antibodies against NCRs (NKp46-clone 9E2, NKp44-clone P44-8, NKp30-clone P30-15), CD16 (clone 3G8), (Biolegend, CA), DNAM-1, and NKG2D (R&D systems, Germany) were used in neutralization assays at 10 μg/ml concentration. Mouse IgG1 (MOPC-21-Biolegend, CA) was used as an isotype control. Antibodies against NKG2D ligands (MICA/B and ULBP1/2) were a kind gift from A. Cerwenka, DKFZ, Heidelberg). Antibodies against DNAM1 ligands (PE-coupled CD155 and CD112) were purchased from Biolegend, CA. Antibodies against CD40 (clone HI40a), CD80 (clone MEM-233), CD86 (clone BU63), and MHC class II (clone MEM-12) were purchased from Immunotools (Friesoythe, Germany). For FACS staining, cells were suspended in 50 μl FACS buffer (PBS, 2% FCS) and incubated with antibodies for 20 min on ice. All washing steps were performed with cold FACS buffer. The cells were then immediately analyzed on a FACS Scan (BD Biosciences, Heidelberg, Germany).

To investigate the expression of NCR ligands, we used recombinant human NKp30-IgGfc, NKp44-IgGfc, and NKp46-IgGfc fusion proteins (R & D Systems, Minneapolis, MN). Staining of NCR ligands was performed by adding 5 μl reconstituted IgG fc fusion protein to 3 × 10^5^ cells in a 100-μl volume and incubating for 2 h on ice without blocking. After washing with cold PBS, the cells were incubated with FITC-conjugated goat anti-human IgG fcγ secondary antibody (Jackson ImmunoResearch) for 30 minutes in the dark. The cells were then washed and used for FACS analysis.

### Cytotoxicity assay

Target cells grown to mid-log phase (5 × 10^5^ cells) were labeled for 1 h at 37°C in 100 μl CTL assay medium (RPMI with 10% FCS and penicillin/streptomycin) with 100 μCi ^51^Cr (Perkin Elmer, Germany). Target cells (T) were washed twice and resuspended in assay medium. Effector cells (E) were seeded onto a V-bottom 96-well plate with 5000 target cells/well at different E:T ratios and, after a 2-min low-speed centrifugation, incubated at 37°C for 4 h in the presence of 100 IU/ml IL-2. Maximum release was determined by treating target cells with 1% Triton X-100 (Sigma, Germany). For spontaneous release, targets were incubated without effectors in assay medium alone. In inhibition assays, effector cells were incubated with mouse anti-human NKG2D, CD16, NCRs, or DNAM-1 antibodies or with the mouse isotype MOPC-21 at 10 μg/ml final concentration for 1 h before mixing with target cells. All samples were tested in triplicate. Supernatants were harvested and ^51^Cr release was measured in a gamma counter. The percentage of specific release was calculated as follows: (experimental release-spontaneous release)/(maximum release-spontaneous release) × 100.

### Ethics statement

Primary human NK cells were isolated from buffy coats purchased from the Institute for Clinical Transfusion Medicine and Cell Therapy, Heidelberg. The Ethics Committee of the University of Heidelberg permitted the use of buffy coats for research purposes without the informed consent of the anomymous blood donors.

### Results

#### IL-2-activated NK cells effectively target colon carcinoma cells

**Figure 1:**
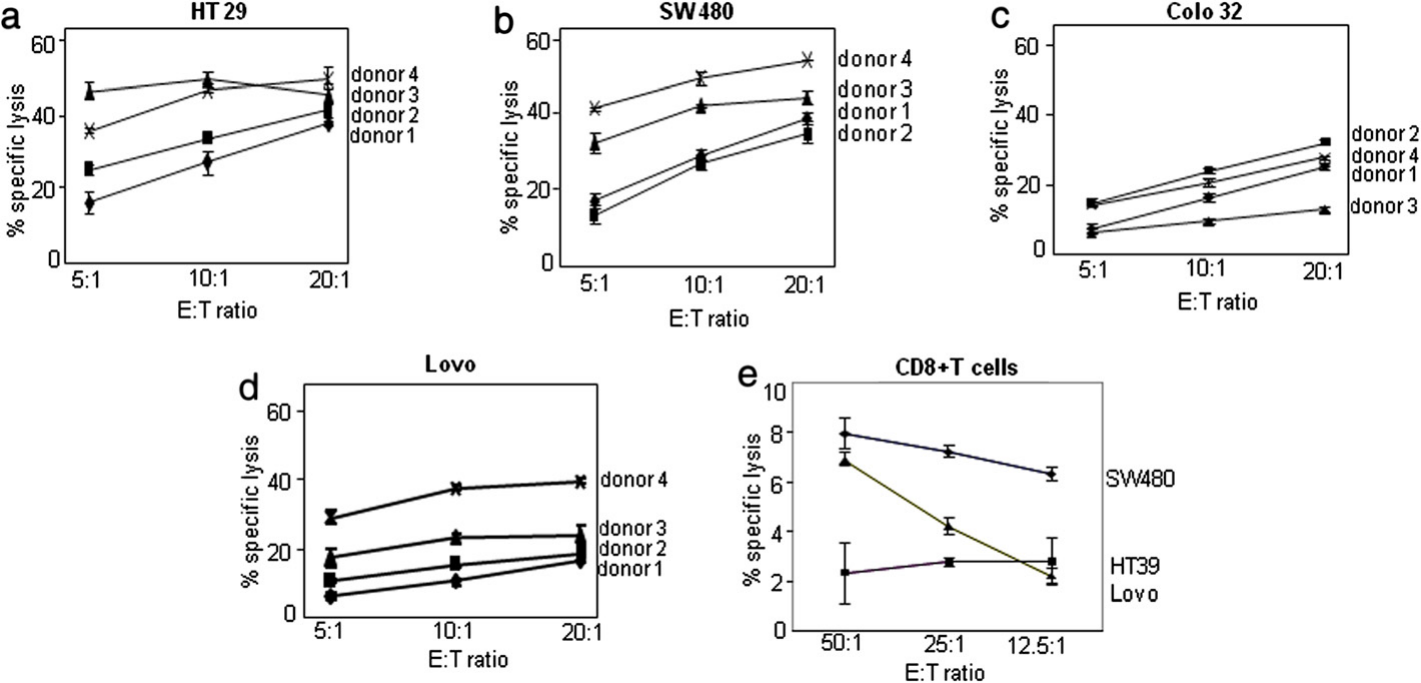
**Killing of colon carcinoma cells by IL-2 -activated NK cells and CD8+T cells.** Freshly isolated NK cells **(**Figure 1**a**-**d)** and CD8+ T cells **(**Figure 1**e)** were cultured with IL-2 (100 U/ml) for one week and used as effector (E) cells in 4-h ^51^Cr release assays performed on the HT29 **(**1**a)**, SW480, **(**1**b)** Colo32 **(**1**c)**, and Lovo **(**1**d)** cell lines. NK-cell-mediated cell lysis was measured at the indicated E:T ratio. Data are means (with SD bars) of triplicates performed with NK cells from one donor. They are representative of independent experiments performed with material from three different donors.

*In vitro*^51^Cr release assays were performed to test the (heretofore unknown) capacity of NK cells to kill colon carcinoma cells. NK cells isolated from healthy donors were stimulated with IL-2 (100 IU/ml) for a week or two and used as effector cells against ^51^Cr-labeled target colon carcinoma cells in a 4-h incubation assay. Using an E:T ratio ranging from 5:1 to 20:1, we found NK cells to be potent killers of colon carcinoma cells under these experimental conditions. In assays performed with NK cells from the same donors, the tested cell lines displayed differential susceptibility to killing by NK cells. SW480 and HT29 cells proved to be the most susceptible (Figure 1a, b), whereas Lovo and Colo32 cells showed low to moderate susceptibility (Figure 1c, d). Alloreactive cytotoxic CD8+ T cells from different healthy donors were likewise stimulated with IL-2 (100 U/ml) and tested for cytotoxicity towards colon carcinoma cells. These cells showed only a low level of colon carcinoma cell killing (Figure 1e). We conclude that IL-2-activated NK cells may constitute an effective tool for targeting colon carcinoma cells.

### H-1PV treatment of colon carcinoma cells increases NK-cell-induced killing

**Figure 2:**
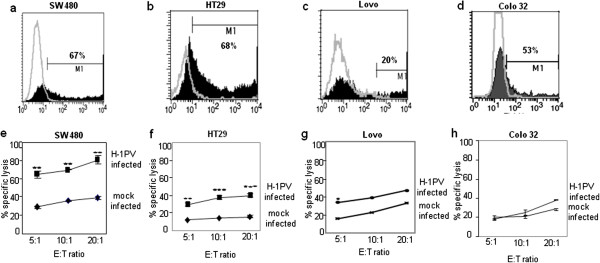
**Efficiency of H-1PV infection and NK-cell-mediated killing of mock- and H-1PV-infected colon carcinoma cells.** Colon carcinoma cells were buffer-treated or infected at MOI=5 RU/cell with recombinant H-1PV expressing the marker EGFP (Chi-hH1/EGFP). The proportion of cells expressing EGFP was determined by flow cytometry. Graphs **a**-**d**: gray lines: autofluorescence profiles of mock-treated cells; black columns: specific staining profiles of Chi-hH1/EGFP-infected. Graphs **e**-**h**: Colon carcinoma cells were infected with H-1PV (MOI=5RU/cell) or mock-treated, incubated for 24 h, and labeled with ^51^Cr for 1 h. Labeled cells were then incubated with IL-2-activated NK effector cells (E) for 4 h at the indicated E:T ratio, and cell lysis was measured (Figure 3**e**-**h**). The data shown are means with SD bars of the results corresponding to NK cells from 4 different donors, each measurement being performed in triplicate.

As oncolytic viruses appear as novel therapeutic tools against cancer and as they can have immunomodulating activities, we examined whether H-1PV might modulate the action of NK cells against colon carcinoma cells. Having previously demonstrated that H-1PV infection of pancreatic carcinoma cells, in addition to causing oncolysis, leads to upregulation of the cytotoxicity of cocultured NK cells [11], we examined how this parvovirus might affect colon carcinoma cells. Colon carcinoma cell cultures were infected for 24 h at MOI = 5 RU/cell with a recombinant H-1PV transducing the enhanced green fluorescent protein (Chi-hH1/EGFP). At the end of the incubation period, the proportion of EGFP-positive cells was determined by FACS as a measure of the infection efficiency (Figure 2a-d). In this analysis, the infection efficiency proved to be higher for the SW480 and HT29 cell lines (Figure 2a, b) than for the Lovo and Colo32 cell lines (Figure 2c, d).

We then examined whether H-1PV infection of colon carcinoma cell lines might increase their susceptibility to killing by IL-2-stimulated NK cells. The colon carcinoma cell lines were infected with H-1PV (MOI=5 RU/cell) or mock-treated for 24 h, labeled with ^51^Cr, and co-incubated with IL-2-stimulated NK cells at different E:T ratios for 4 h. Using NK cells from several different donors, we found H-1PV-infected SW480 cells to be killed by NK cells at a significantly higher rate than mock-infected cells (Figure 2e). Similar results were obtained with HT29 and Lovo cells (Figure 2f, g). The increase in Colo32-cell death was minimal (Figure 2h). No virus-mediated lysis of H-1PV-infected target cells was seen on day 1 post-infection or after 4 h of incubation in the killing assays. Altogether, these data suggest that upon H-1PV treatment, colon carcinoma cells probably undergo phenotypic changes rendering them more susceptible to killing by NK cells.

### Natural cytotoxicity receptors mediate killing of colon carcinoma cells by NK cells

**Figure 3:**
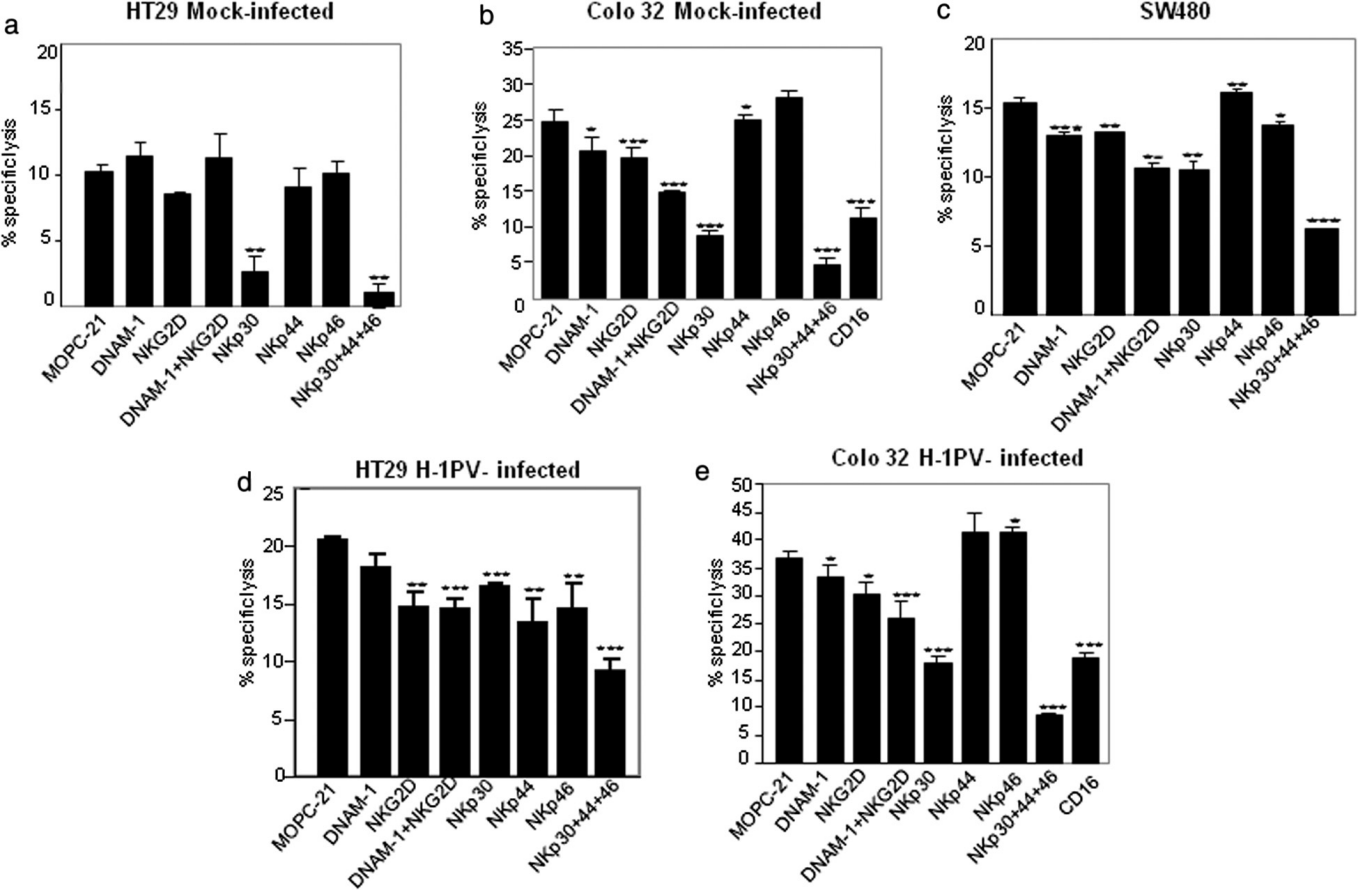
**Role of NK-cell receptors in killing mock- and H-1PV-infected colon carcinoma cells.** HT29 and Colo 32 cells **(d**-**e)** were infected with H-1PV (MOI = 5 RU/cell) or mock-treated **(a**-**b)** for 24 h and used as targets (T) for IL-2-activated NK effector (E) cells (E:T=10:1) in 4-h chromium release assays. Prior to their co-incubation with target cells, the effector cells (E) were incubated for 1h with 10 μg/ml blocking antibodies specific to the indicated receptor or with the unrelated MOPC-21 IgG1 isotype. Figure 3**c** shows results from uninfected SW480 cells. Data are expressed as percentages of target cell killing and represent means (with SD bars) of triplicate values from one representative donor. The statistical significance of differences in target-cell lysis was calculated with Student’s *t* test (*p, <0.05; **, p<0.01; ***, p<0.001).

Having shown that NK cells are potent killers of colon carcinoma cells, we investigated the mechanism of this killing. For this we incubated IL-2-activated NK cells with antibodies targeting various receptors before estimating the killing effect in 4-h ^51^Cr release assays. Inhibition of killing was measured as the percent decrease in cell lysis resulting from blocking of the tested receptor. All the results were compared to a control using unrelated MOPC-21 IgG1 isotype. The results highlighted natural cytotoxicity receptors (NCRs) as the major receptors involved in killing of colon carcinoma cells (Figure 3a-e). Blocking of the NKp30 receptor alone was found to inhibit killing of mock-infected HT29 cells quite strongly and that of H-1PV-infected cells to a lesser extent. Combined blocking of the NKp30, NKp44 and NKp46 receptors proved most effective at inhibiting killing of both mock- and H-1PV-infected HT29 cells. Similar results were obtained with mock- and H-1PV-infected Colo32, and SW480 cells (Figure 3b, c). These observations prove that the NCRs- NKp30, NKp44, and NKp46 acting together play a major role in mediating the cytotoxicity of NK cells towards mock- and H-1PV-infected colon carcinoma cells. Blocking of the NKG2D or NKp44 receptor alone also decreased killing to some extent (Figure 3a, d). Since it has been shown that CD16 mediates direct NK cell cytotoxicity, in addition to antibody-dependent cell-mediated cytotoxicity [15], we tested test whether CD16 receptor blocking could also modulate Colo 32 cell lysis by NK cells in our experimental settings under the same assay conditions as for other blocking antibodies. We observe that CD16 inhibition also decreased killing of mock and H-1PV infected Colo32 cells but less as compared to combined NCR blocking (Figure 3b, e).

### H-1PV infection modulates surface ligand expression on colon carcinoma cells

**Figure 4:**
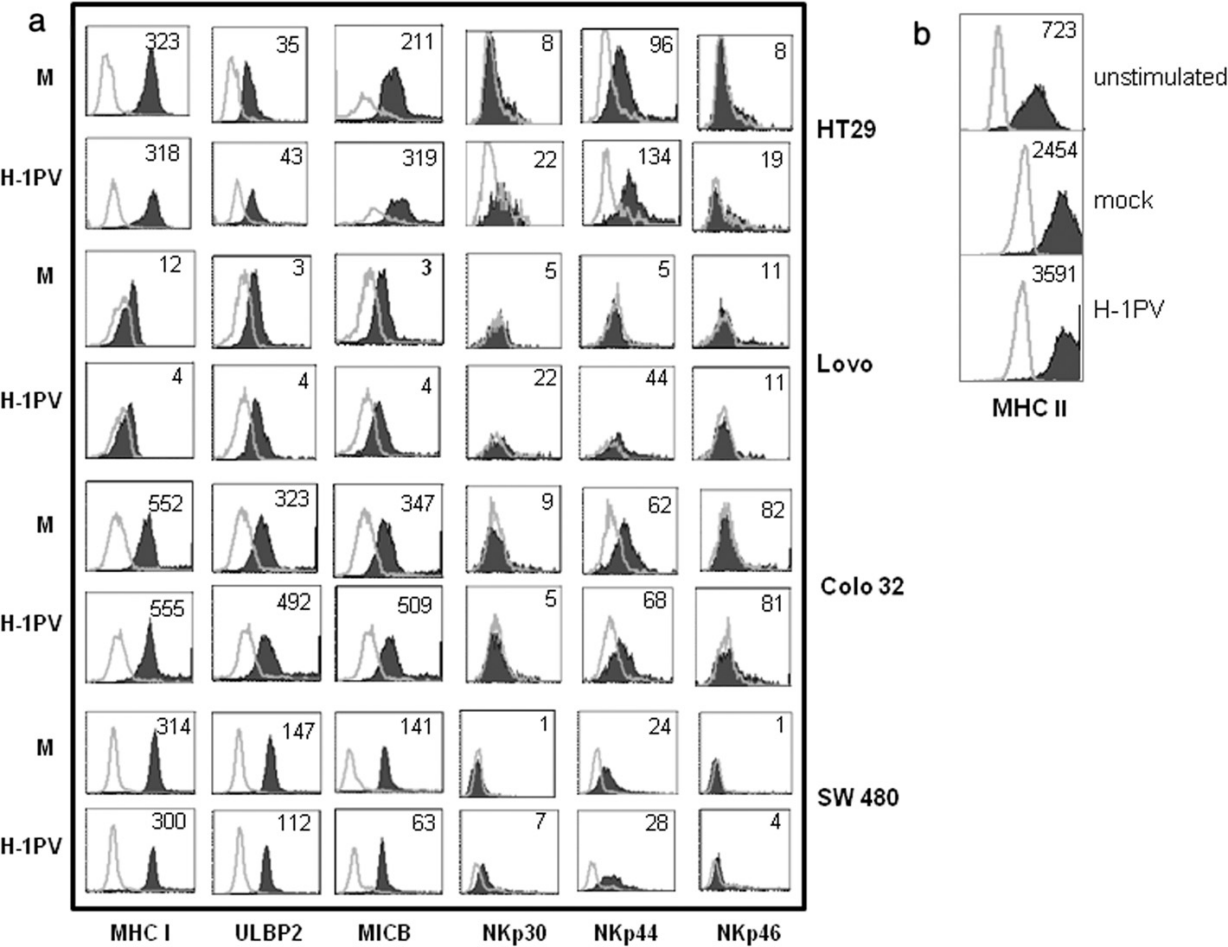
**Effect of H-1PV infection on the phenotype of colon carcinoma and dendritic cells. (a)** Colon carcinoma cells were buffer-treated (M) or H-1PV-infected (MOI=5 RU per cell), incubated for 24 h, and analyzed by flow cytometry for expression of MHC class I, MICB, and ULBP 2 molecules and NCR (NKp30, NKp44, and NKp46) ligands. Control mouse IgG and specific antibody staining profiles are shown by gray lines and black columns, respectively. The indicated values represent ∆MFI=MFI (positive)-MFI (isotype/negative control) for one representative experiment out of three. **(b)** Colo32 cells were mock-treated (M) or H-1PV-infected (MOI= 5RU/cell) and lysates prepared on day 1 p.i. Dendritic cells were then pulsed with lysate for 2days and thereafter, analyzed for expression of MHC class II molecules, and compared with untreated dendritic cells. Figure 4**(b)** shows the means of data obtained from 3 donors. Control mouse IgG and specific antibody staining profiles are shown by grey lines and black columns, respectively. The indicated values represent ∆MFI = MFI (positive)-MFI (isotype/negative control).

To investigate the mechanism by which H-1PV infection enhances NK-cell-induced killing of colon carcinoma cells, we analyzed the expression of different ligands and of MHC class I molecules on mock- and H-1PV-infected cells. Upon H-1PV infection, MHC class I expression was found to be down regulated on Lovo cells but unchanged on the other colon carcinoma cells tested. Neither the tested NKG2D ligands (ULBP1 and MICA, data not shown; ULBP2 and MICB, Figure 4a), nor the tested DNAM1 ligands (CD155 and CD112, data not shown) showed any upregulation. Our finding that NCRs are involved in NK-cell-induced killing of colon carcinoma cells prompted us to test mock- and H-1PV-infected colon carcinoma cells for expression of NCR ligands, using NKp30-IgGFc, NKp44-IgGFc, and NKp46-IgGFc fusion proteins for ligand binding and a secondary antibody to detect the Fc. Colon carcinoma cells showed moderate expression of NKp44 ligands and low expression of NKp30 and NKp46 ligands. To determine the effect of H-1PV infection on NCR ligand expression, we infected the colon carcinoma cells with H-1PV at MOI = 5 and analyzed the cells on day 1 post infection. Upon H-1PV infection, HT29, Lovo, and SW480 but not Colo32 cells, displayed several fold increase in NKp30 ligand expression. Lovo cells showed an increase in NKp44 ligand expression after H-1PV infection. HT29 cells exhibited a two-fold increase in NKp46 ligand expression (Figure 4a).

After this phenotypic assessment of mock- and H-1PV-infected colon cancer cells, we investigated whether lysates of H-1PV-infected colon carcinoma cells might influence the phenotype of human dendritic cells. Monocyte-derived dendritic cells were pulsed for 2 days with 50 μg lysate of mock- or H-1PV-infected Colo32 cells (MOI = 5 pfu/ml). The lysates were prepared by repeated freezing/thawing of mock- and H-1PV- infected cells. The dendritic cells were then analyzed by flow cytometry for surface expression of CD40, CD80, CD86, and MHC class II. We failed to detect any change in CD40, CD80, or CD86 expression on dendritic cells treated with either lysate (data not shown), but MHC class II expression was increased, as compared to untreated cells, when the cells were treated with lysate of mock-infected Colo32 cells, and a greater increase was observed upon treatment with lysate of H-1PV-infected cells (Figure 4b). Altogether, our results demonstrate that a lysate of H-1PV-infected cells can upregulate MHC Class II expression on dendritic cells.

## Discussion

There is growing interest in exploring the potential of NK cells in cancer. It appears, however, that during tumor development, NK cells are in a state of suppression. Cytokines can be used to enhance NK-cell antitumor activity. Upon activation with cytokines, particularly IL-2, NK cells can be activated *in vitro* to exert potent cytotoxicity against tumors [16]. Here we show that IL-2-activated NK cells can effectively kill colon carcinoma cells, although the susceptibility of these cells is variable.

Novel anticancer therapies based on oncolytic viruses are also emerging. In particular, the oncolytic parvovirus H-1PV appears as a promising tool for developing such strategies. A major advantage of this virus is that normal human cells, as opposed to certain cancer cells, are refractory to H-1PV infection. Yet there is an obstacle to exploiting the oncolytic properties of H-1PV in the framework of colon carcinoma: colon carcinoma cells show H-1PV-triggered lysis only at high MOI and after a long incubation period, and the virus shows impaired replication in these cells [9,10]. This is why we have focused on another property of H-1PV: its ability to enhance killing of cancer cells by NK cells. This is the first study to examine this property in colon carcinoma cell models. We clearly demonstrate that H-1PV infection renders colon carcinoma cells more vulnerable to killing by NK cells.

To understand the molecular mechanism of NK cell cytotoxicity, it is necessary to understand the interaction between NK cell receptors and tumor ligands [17]. We show here that the natural cytotoxicty receptors NKp30, NKp44, and NKp46, unique to NK cells, are involved in killing the colon carcinoma cell lines tested. As shown in our experiments where these receptors were blocked, the combined interaction of these NCRs is primarily responsible for NK-cell-triggered lysis of both mock- and H-1PV-infected colon carcinoma cells. This suggests that the cellular ligands of NCRs, though still elusive, are expressed on colon carcinoma cells. Our FACS analysis of NCR ligand expression shows that NCR ligands are present at low to moderate levels on colon carcinoma cells, but that H-1PV infection causes several fold increase in NCR ligand expression. This could account for the increased killing by NK cells observed upon H-1PV infection. Even though the upregulation of individual NCR ligands is limited, it could lead to cumulative activation of the NK-cell killer effect. Lovo cells, furthermore, showed downregulation of MHC I expression upon H-1PV infection. This, in addition to NCR ligand upregulation, could lead to increased susceptibility of these cells to killing by NK cells. Colo32 cells, in contrast, show no increase in NCR ligand expression upon H-1PV infection. This may explain why H-1PV-infected Colo32 cells show only minimally increased killing by NK cells. It is important to mention here that the low level of NKp30 ligand expression in colon carcinoma cells does not correlate with the high dependence of NK cell lysis on NKp30 receptor as shown in antibody-blocking assays. We speculate that the lack of correlation could be due to modification of NKp30 ligand(s) in these cells, leading to their altered recognition by the fusion proteins. Alternatively, other unknown soluble ligand(s) may be involved, in agreement with a recent report [18]. This discrepancy has also been reported in other studies [19-21]. Neither DNAM1 nor NKG2D blocking results in significant inhibition of killing of colon carcinoma cells (Figure 3). Worth mentioning is the fact that colon carcinoma cells show high-level expression of the DNAM1 ligands CD155 and CD112 (data not shown) but no upregulation upon H-1PV infection.

Oncolytic virotherapy can release into the tumor microenvironment a wide range of tumor-associated antigens that can be taken up by antigen-presenting immune cells such as dendritic cells, so as to prime T cells and thereby amplify immune responses [22,23]. Parvovirus H-1PV-induced tumor cell killing has been shown to promote cytotoxic T lymphocyte responses through increased phagocytosis, maturation, and cross-presentation by dendritic cells [24]. Likewise, significant activation of dendritic cells and microglia has been observed upon incubation with parvovirus-MVM-infected glioma cells [25]. Here have found only a limited effect of H-1PV on the phenotype of dendritic cells, as only MHC class II expression was upregulated upon exposure to H-1PV-infected Colo32-cell lysates. We chose Colo 32 cell-line as these cells are relatively sensitive to H-1PV infection as compared to other colon carcinoma cells lines [9,10]. No such effect was observed with H-1PV-infected Lovo- or HT29-cell lysates. Furthermore, the effect was observed only when 50 μg lysate was used to treat the dendritic cells. This suggests that optimization of the lysate concentration is important in devising strategies for immunotherapy.

The present data on the effects of H-1PV on colon carcinoma cells are consistent with and extend our previous data showing NK cell activation in response to H-1PV infection of pancreatic cancer cells. It is noteworthy that under identical experimental conditions used in this study, H-1PV infection of normal IL-2 stimulated human PBMCs failed to activate NK cells as measured through IFN-γ release [11]. This argues for the tumor specificity of NK cell sensitization. These data are also in line with data on other oncolytic viruses, such as reoviruses and Newcastle disease virus, shown to stimulate innate and adaptive antitumor activity [22,23]. Here we demonstrate that upon infection of colon carcinoma cells, H-1PV can cause neighboring NK cells to exert antitumor activity, even though the virus’s direct oncolytic effect on these cells is small. Although the mechanism of action of the parvovirus remains to be investigated, our results may have therapeutic relevance in the colon carcinoma context. Cytokines, particularly IL-2, are known to be indispensable to the regulation of NK-cell cytotoxicity. Previously, we have demonstrated in a nude-mouse pancreatic cancer model that the recombinant H-1PVs encoding IL-2 or the chemokine MCP-3/CCL7 cause recruitment of activated NK and monocytes to the site of tumor, resulting in a strong antitumor response [26]. It would be interesting to evaluate the efficacy of cytokine-encoding particularly IL-2-encoding and chemokine-encoding) recombinant H-1PVs in a colon cancer model, too. Recently, variants of IL-2 have been developed that caused robust expansion and activation of cytotoxic CD8^+^ T cells and NK cells but limited expansion of Treg cells, thereby mitigating adverse effects associated with wild type IL-2 [27]. Arming of recombinant H-1PV with these novel IL-2 variants or supplementing H-1PV-based virotherapy with IL-2 ‘superkine’ is worth considering to improve the efficiency of tumor therapy.

## Conclusions

In conclusion, this study shows that infection of colon carcinoma cells with H-1PV is immunogenic, causing activation of innate immune cells in contact with infected cancer cells. This adjuvant effect of the virus can be expected to compensate, at least the poor oncolytic effect of H-1PV on colon carcinoma cells, and to contribute to oncosuppression. This opens hopeful prospects for the development of parvovirus-based virotherapies of colon carcinoma.

## Abbreviations

NK: Cells: natural killer cells; RU: Replication unit; PV: Parvovirus; IL-2: Interleukin-2

## Competing interests

The authors declare that they have no financial or non-financial competing interests.

## Authors’ contributions

RB designed the study, performed the experiments, and wrote the paper. JR critically revised and finally approved the version to be published. Both authors read and approved the final manuscript.

## Pre-publication history

The pre-publication history for this paper can be accessed here:

http://www.biomedcentral.com/1471-2407/13/367/prepub
